# CAD-RADS 2.0: Comparison of methods for assessment of plaque burden

**DOI:** 10.1016/j.ejro.2026.100752

**Published:** 2026-04-20

**Authors:** Ivan Platzek, Krzysztof Nocon, Suela Mema, Ralf-Thorsten Hoffmann, Axel Linke, Felix Matthias Heidrich, Stefanie Jellinghaus

**Affiliations:** a Dresden University Hospital, Department of Radiology, Dresden, Germany; b Dresden Heart Center, Department of Cardiology, Dresden, Germany

**Keywords:** Coronary computed tomography angiography, Plaque burden, Coronary Artery Disease-Reporting and Data System, Method agreement, Segment involvement score

## Abstract

**Objectives:**

To assess the agreement of the three methods of coronary plaque burden assessment approved by the Coronary Artery Disease-Reporting and Data System classification (CAD-RADS 2.0): coronary artery calcium (CAC) testing, segment involvement score (SIS), and visual estimate.

**Methods:**

Patients with stable chest pain who underwent coronary computed tomography angiography (CCTA) and showed signs of coronary artery disease were included in the current retrospective study. The CCTAs were independently evaluated by two board-certified radiologists. Differing results were resolved in consensus. Three separate estimates of plaque burden (P) were performed for each examination: P(CAC), P(SIS) and P(visual). Linearly weighted kappa was used to assess the agreement of the three methods.

**Results:**

The study included 132 patients (mean age 66.6 years, 68.2% male). The mean CAC score was 503.6 ± 1023.5 [SD]. CAD-RADS was 1 in 47/132 patients (35.6%), 2 in 40/132 patients (30.3%), 3 in 18/132 patients (13.6%), 4 in 24/132 cases (18.2%) and 5 in 3/132 cases (2.3%). Weighted Kappa values for method agreement were κ= 0.56 (95% CI: 0.48–0.65) for agreement between P(CAC) and P(SIS), κ= 0.67 (95% CI: 0.59–0.75) for agreement between P(CAC) and P(visual), and κ= 0.72 (95% CI: 0.64–0.80) for agreement between P(SIS) and P(visual), signifying moderate to substantial agreement.

**Conclusion:**

Results for plaque burden in CAD-RADS 2.0 are clearly influenced by the choice of assessment method. When reporting plaque burden in CAD-RADS 2.0, the method of assessment should be unequivocally named, as the three proposed methods are not interchangeable and describe different aspects of plaque burden.

## Highlights

• The methods for coronary plaque burden assessment allowed in CAD-RADS 2.0 show moderate to substantial agreement. .
• Interrater agreement for the segment involvement score (SIS) and visual plaque estimate is high.
• Overall CAD-RADS 2.0 categories show a high degree of interrater agreement.

## 1 Introduction

Coronary computed tomography angiography (CCTA) offers a unique possibility to assess both the lumen and the wall of coronary arteries noninvasively. The high sensitivity of the method allows for the reliable exclusion of coronary artery disease as well for the detection of subclinical atherosclerosis. Originally introduced in 2016, the Coronary Artery Disease-Reporting and Data System (CAD-RADS) is now widely used for standardized CCTA reporting. Its current version, CAD-RADS 2.0, introduced a plaque burden sub-classification (P), which allows for values between P1 (mild amount of plaques) and P4 (extensive amount of plaques). The method for classifying plaque burden in CCTA is not unified. Three different methods for assessing plaque burden are allowed in CAD-RADS 2.0: coronary artery calcium (CAC) testing, segment involvement score (SIS), and visual estimate.

This decision reflects differing opinions about the value of CAC testing and also takes into account that the time available to read the CCTA scans may differ between sites. However, allowing alternative methods of plaque quantification also may introduce a systematic error. The alternative paths of plaque quantification are only meaningful if they show good agreement, which has not been evaluated yet.

The quantified plaque burden may have direct therapeutic implications, particularly in the context of secondary prevention, as lipid-lowering therapy is strongly recommended [1]. The treatment goal for LDL-C reduction should be guided by a combination of clinical risk factors and the extent of plaque burden. A currently published Danish cohort study demonstrated that non-obstructive but extensive atherosclerosis is associated with a statistically higher incidence of all-cause mortality or myocardial infarction compared to non-obstructive, non-extensive disease [2], underscoring the need for standardized plaque burden quantification in risk stratification and clinical decision-making.

In patients with stable chest pain, CAD-RADS 2 and P3 or 4, the original CAD-RADS 2.0 consensus paper recommends “aggressive risk factor modification and preventive pharmacotherapy.” For comparable patients, but with a lower plaque burden classification of P1 or P2, the recommendation is less urgently formulated as “risk factor modification and preventive pharmacotherapy.”

The aim of this study was to assess the agreement of the methods of coronary plaque burden assessment approved by CAD-RADS 2.0.

## 2 Methods

### 2.1 Study design and population

This retrospective monocentric study was approved by the ethics committee of Dresden University Hospital (reference EK-98022025). The radiology information system (RIS) of our hospital was searched for patients with stable chest pain who underwent coronary computed tomography angiography (CCTA) between October 2023 and January 2024.

Inclusion criteria were CCTA, including a non-enhanced scan for CAC testing, age over 18 years, absence of coronary stents, a signed patient consent form for CCTA data to be used in research, and signs of coronary artery disease on CCTA. The patients were only included if all the criteria mentioned above were fulfilled.

### 2.2 CCTA acquisition

All examinations were performed on a third-generation dual-source CT system (Somatom Force, Siemens Healthineers, Erlangen, Germany). This CT scanner utilizes two x-ray tubes with an angular offset of 90°, with two separate corresponding detectors (192 slices each). It allows for three acquisition protocols for CCTA: step-and-shoot (SAS) mode with prospective ECG gating, prospective ECG-gated high-pitch spiral, and a low-pitch spiral with retrospective ECG gating [3].(Table 1)

**Table 1:** Patient and CT scan characteristics.

Patient Characteristics	
Total patients	132
Male	42 (31.8%)
Female	90 (68.2%)
Mean age (y)	66.6 ± 10.2 [SD]
Mean body mass index	26.7 ± 3.7 [SD]
Mean heart rate	67 ± 12 [SD]
Scan characteristics	
Mean scan length/CAC scan (mm)	128.2 ± 19.3 [SD]
Mean dose-length product (DLP)/CAC scan (mGy.cm)	36.5 ± 46.3 [SD]
Mean effective dose/CAC scan (mSv)	0.7 ± 0.8 [SD]
Mean scan length/contrast-enhanced CCTA (mm)	118.7 ± 14.67 [SD]
Mean dose-length product (DLP)/contrast-enhanced CCTA scan (mGy.cm)	240.9 ± 271.2 [SD]
Mean effective dose/ contrast-enhanced CCTA (mSv)	4.3 ± 6.7 [SD]

The choice of the acquisition protocol depended on heart rate, the presence of cardiac arrhythmias, and body habitus. Protocols with prospective ECG gating were used in patients with a heart rate lower than 70/min and a regular sinus rhythm.

The patients had to abstain from solid food for 4 h before the CCTA examination. They also had to refrain from taking PDE-5 inhibitors such as sildenafil for 48 h before the CCTA [4] and were instructed to refrain from caffeine for 12 h before the examination.

All patients were examined in the supine position. The blood pressure and heart rate of the patients were measured before deciding on the need for premedication.

In patients with a heart rate higher than 70/min, metoprolol (Metoprolol Carinopharm®, Carinopharm GmbH, Eime, Germany) was applied intravenously to reduce it. This excluded patients with contraindications for the use of beta-blockers, including asthma, atrioventricular block, acute heart failure, and hypotension with systolic pressures < 90 mm Hg. Depending on initial heart rate and the patients’ reaction to the initial metoprolol injection, the total dose of metoprolol varied between 2.5 and 15 mg [5].

All patients without contraindications for nitrates received a single push of sublingually applied glycerol trinitrate (Nitrolingual™, Pohl-Boskamp, Hohenlockstedt, Germany) before the scan. Contraindications for nitrates were hypotension with systolic pressures < 90 mm Hg, aortic stenosis, hypertrophic cardiomyopathy, and known intolerance to nitrates.

The scan range of the CT examination was between the inferior border of the tracheal bifurcation and the inferior border of the heart.

A nonenhanced CAC scan was acquired using a high-pitch protocol in all patients, with a uniform tube voltage of 120 kV and adaptive tube current. In this case the collimation was 2 × 192 × 0.6 mm, and the pitch (the ratio of table travel per gantry rotation to the overall thickness of the simultaneously acquired slices) was 3.2.

50 ml of iohexol (Accupaque™ 350, GE Healthcare, Chicago, Ill, USA) was applied intravenously via an 18 G intravenous catheter placed in an antecubital vein at a flow rate of 5 ml/s, followed by 50 ml of saline at the same flow rate, using a dual-head injector (MEDRAD Stellant, Bayer AG, Leverkusen, Germany).

The bolus tracking technique was used to start the acquisition of the contrast-enhanced CCTA scan and assure optimal contrast filling of the coronary vessels. The region of interest (ROI) for bolus tracking was placed in the ascending aorta. The trigger threshold was set at 120 Hounsfield Units (HU). The CCTA scan started 5 s after the trigger threshold was reached. All CCTA scans were performed in inspiration.

The step-and-shoot mode used a collimation of 2 × 152 × 0.6 mm, while the high-pitch spiral used a collimation of 2 × 192 × 0.6 mm and a pitch of 3.2. The low-pitch spiral had a detector collimation of 2 × 192 × 0.6 mm and a pitch of 0.15. In the case of the low-pitch spiral, additional ECG pulsing was used to reduce the radiation exposure, with the ECG-pulsing window set between 30% and 80% of the R–R interval.

The scan was performed in the craniocaudal direction in all patients. Tube voltage and tube current of the CCTA scan were adapted by the scanner software CareDose4D (Siemens Healthineers) depending on the physique of each patient. CCTA images were reconstructed with a slice thickness of 0.6 mm using a medium smooth kernel (Bv40) and Advanced Modeled Iterative Reconstruction [6] (ADMIRE) level 3 (out of five possible ADMIRE levels provided by the CT scanner).

### 2.3 Radiation exposure evaluation

The effective doses of both the CAC scan and the CCTA (in mSv) were calculated by multiplying the dose-length products provided by the scanner with a conversion factor of 18 µSv/mGycm as recommended by Huda [7].

### 2.4 CCTA evaluation

The CCTAs were independently evaluated by two board-certified radiologists, with 12 and 4 years of experience with CCTA, respectively. Each reader was blinded to the findings of the other reader and to other imaging studies of the patients.

Each reader performed three separate readings of the CCTA examinations, each separated by two weeks to avoid bias. In the initial reading, the total CAC score was automatically calculated according to the Agatston method [8] by software provided by the CT vendor (Syngo Via VB 40, Siemens Healthineers, Erlangen, Germany).

In this initial reading, the overall CAD-RADS category for each patient was determined by each radiologist according to CAD-RADS 2.0 [9], ranging from 0 (no plaque or stenosis) to 5 (total occlusion).

In the two later readings, the radiologists only assessed the plaque burden for each scan based on the segment involvement score (SIS) and on visual estimate, respectively, following the criteria formulated in the CAD-RADS 2.0 consensus paper [9].

The summary segment involvement score and visual assessment score were determined in consensus by the two readers.

Subsequently, P scores based on CAC testing, SIS, and visual assessment were calculated for each patient, called P(CAC), P(SIS), and P(visual). As described in the CAD-RADS 2.0 consensus paper, P can have whole values between 1 and 4. A value of 0 was not originally envisaged, as CAD-RADS 0 is synonymous with absence of plaques. However, in order to perform a comprehensive analysis, we assigned a P score of 0 to patients with no coronary plaques (CAC = 0, SIS = 0, or inconspicuous coronary arteries based on visual estimate).

### 2.5 Statistical analysis

Statistical evaluation was performed using RStudio [10] (Posit PBC, Boston, Mass., USA). RStudio is an integrated development environment for the R programming language [11] and supports a great variety of software packages for additional statistical functions not provided by the base program. In this study, two such software packages were used: irr and vcd.

The irr package [12] allows one to determine different measures of method and interrater agreement, including weighted kappa. The vcd package [13] is used for visualization of categorical data, including the agreement charts described below.

In the current study, linearly weighted kappa (κ) was used to quantify interrater agreement for CAD-RADS, SIS, and the visual assessment of plaque burden [14], [15]. Linearly weighted kappa was also used to assess the agreement of P scores for the three different methods mentioned above: CAC, SIS, and a visual assessment score. Weighted kappa values were interpreted according to the criteria proposed by Landis and Koch [16] (κ ≤ 0 poor agreement; 0.01–0.20 slight agreement; 0.21–0.40 fair agreement; 0.41–0.60 moderate agreement; 0.61–0.80 substantial agreement; and 0.81–1.00 almost perfect agreement).

The agreement chart proposed by Bangdiwala [17] was used to visualize pairwise agreement between the different methods for plaque burden evaluation. These agreement charts use a coordinate system with an x- and y-axis. In our case, each axis represents one of the methods being compared. The length of the axis corresponds to the number of observations. Each category (plaque burden category from P = 0 to P = 4 in this case) is represented in the chart by a rectangle whose dimensions depend on the number of observations by each method. The blackened part of each rectangle indicates complete agreement between the two methods. In contrast, gray surfaces represent partial agreement, i.e., a disagreement of just one category [17].

Continuous variables were represented as the mean ± standard deviation [SD]. Categorical variables were expressed as frequencies and percentages.

## 3 Results

### 3.1 Patient and scan characteristics

In the initial search, 207 consecutive patients with CCTA were found. Four patients were excluded because of coronary stents, and one patient was excluded because of insufficient image quality. The remaining 202 patients were evaluated by two radiologists according to the CAD-RADS 2.0 criteria. 70 patients had no signs of coronary artery disease and were excluded.

132 patients had no signs of coronary artery disease (based on segment involvement score and visual assessment) and were thus included in the plaque burden evaluation: 42 (31.8%) female patients and 90 (68.2%) male patients; mean age 66.6 years ± 10.2 [SD].

The characteristics of the patients included in the study are summarized in Table 2.

**Table 2:** Reclassification table for patients with CAD-RADS 1 and 2 (n = 87), comparing P scores based on calcium scoring (CAC) and on segment involvement score (SIS). Boxes signifying the number of patients with change of suggested recommendation according to CAD-RADS 2.0 (P1 and 2 vs P3 and 4) are marked in red. In this case there were 18/87 cases of change of suggested recommendations (20.6%).

In the study cohort, the prospective ECG-gated step and shoot acquisition protocol was used in 95/132 cases (72.0%). The prospective ECG-gated high-pitch acquisition protocol was used in 14/132 examinations (10.6%), while 23/132 patients (17.4%) were examined using a helical protocol with retrospective ECG gating.

### 3.2 CAD-RADS

Before consensus reading, the two reading radiologists agreed on CAD-RADS in 91/132 cases (68.9%) and differed in 41/132 cases (31.1%), consistent with a weighted kappa of κ = 0.77 (95% CI: 0.70–0.84).

Final CAD-RADS after consensus reading was 1 in 47/132 patients (35.6%), 2 in 40/132 patients (30.3%), 3 in 18/132 patients (13.6%), 4 in 24/132 cases (18.2%) and 5 in 3/132 cases (2.3%).

### 3.3 Plaque burden

The mean CAC score was 503.6 ± 1023.5 [SD]. 10/132 patients (7.6%) had a CAC score of 0. Based on the CAC score, overall plaque burden was classified as P = 0 in 10/132 patients (7.6%), P1 in 54/132 patients (40.1%), P2 in 18/132 patients (13.6%), P3 in 34/132 patients (25.87%), and P4 in 16/132 patients (12.1%).

Segment involvement scores (SIS) of both readers were identical in 58/132 cases (44%) and differed in 74/132 patients (56%), with a resulting weighted kappa of κ = 0.82 (95% CI: 0.78–0.85).

SIS after consensus reading varied between 1 and 13. The resulting P (SIS) was P1 in 46/132 cases (34.8%), P2 in 36/132 cases (27.2%), P3 in 28/132 patients (21.2%), and P4 in 22/132 patients (16.7%).

Both readers had identical results for P (visual) in 106/132 cases (80.3%) and differed in 26/132 (19.7%) cases. The corresponding weighted kappa was κ = 0.82 (95% CI: 0.75–0.89), consistent with almost perfect agreement.

P (visual) after consensus reading was P1 in 62/132 patients (47.0%), P2 in 33/132 cases (25%), P3 in 17/132 cases (12.9%), and P4 in 20/132 patients (15.1%).

### 3.4 Method agreement

**Fig. 1:**
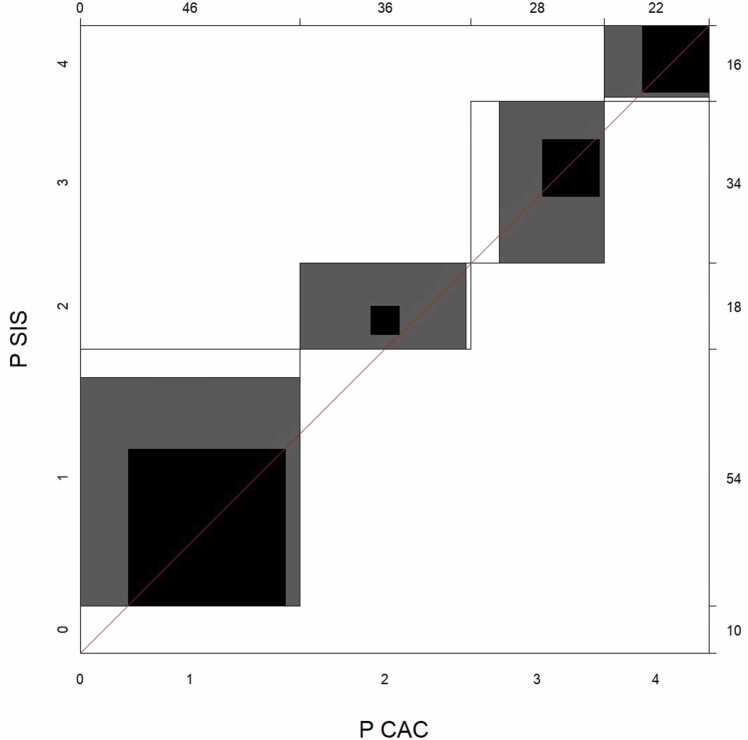
Agreement Chart for the method agreement between plaque burden scores (P) based on SIS (P(SIS)) and CAC testing (P(CAC)).

P(CAC) and P (SIS) were identical in 65/132 patients. P(SIS) was higher than P(CAC) in 47/132 patients and lower in 20/132 patients. The resulting weighted Kappa for method agreement between P(CAC) and P(SIS) was κ= 0.56 (95% CI: 0.48–0.65), signifying moderate agreement. Fig. 1 shows an agreement chart for the method agreement between P(SIS) and P(CAC).

Table 2 presents the agreement of P(SIS) and P(CAC) for patients with CAD-RADS of 1 or 2, the only group in which a change of the P score can lead to a change of suggested clinical recommendations according to CAD-RADS 2.0. When using P(SIS) instead of P(CAC), there were 18/87 cases of change of suggested recommendations (20.6%).

P(CAC) and P(visual) were identical in 87/132 cases (65.9%). When compared to P(CAC), P(visual) was higher in 25/132 cases (18.9%) and lower in 20/132 cases (15.1%).

**Fig. 2:**
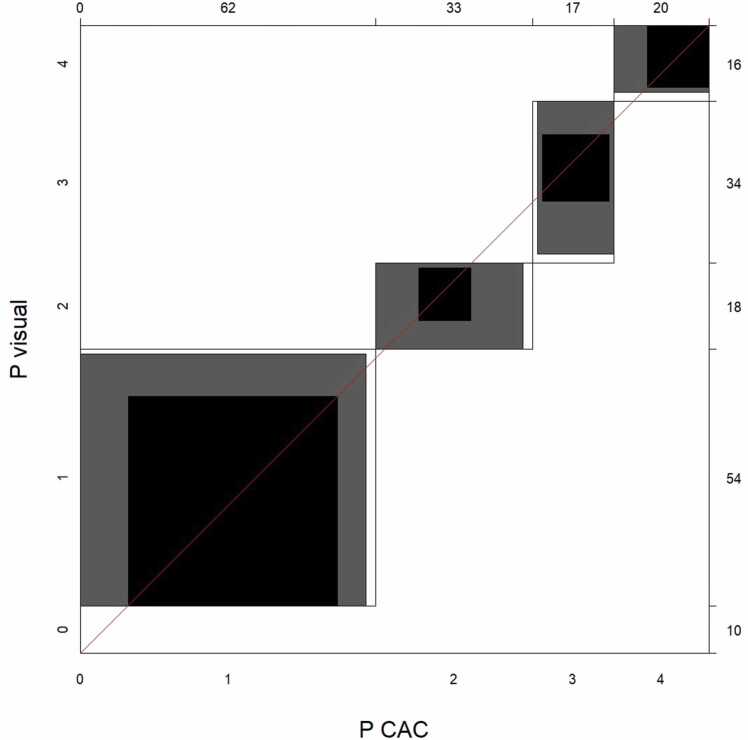
Agreement Chart for the method agreement between plaque burden scores (P) based on CAC testing (P(CAC)) and visual estimate (P(visual)).

The resulting weighted kappa for method agreement between P(CAC) and P(visual) was κ= 0.67 (95% CI: 0.59–0.75), signifying substantial agreement. The corresponding agreement chart for P(CAC) and P(visual) is shown in Fig. 2.

Table 3 presents the agreement of P(CAC) and P(visual) for patients with CAD-RADS 1 or 2. In this subgroup a change of the P score can leads to a change of suggested clinical recommendations according to CAD-RADS 2.0. When using P(visual) instead of P(CAC), there were 3/87 cases of change of suggested recommendations (3.4%).

**Table 3:** Reclassification table for patients with CAD-RADS 1 and 2 (n = 87), comparing P scores based on calcium scoring (CAC) and on visual estimate. Boxes signifying the number of patients with change of suggested recommendation according to CAD-RADS 2.0 (P1 and 2 vs P3 and 4) are marked in red. In this case there were 3/87 cases of change of suggested recommendations (3.4%).

P(SIS) and P(visual) agreed on the extent of plaque burden in 89/132 cases (67.4%) and differed in 43/132 cases (32.6%). P(visual) was higher in 7/132 cases (5.3%) and lower in 36/132 cases (27.3%).

**Fig. 3:**
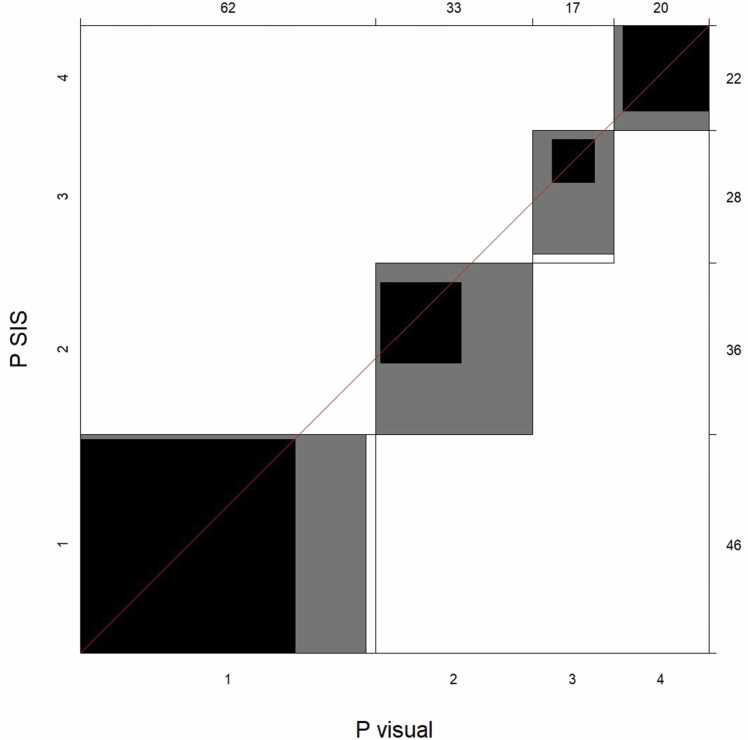
Agreement Chart for the method agreement between plaque burden scores (P) based on SIS (P(SIS)) and visual estimate (P(visual)).

The resulting weighted kappa for method agreement between P(SIS) and P(visual) was κ= 0.72 (95% CI: 0.64–0.80), consistent with substantial agreement. A graphical representation of the agreement between P(SIS) and P(visual) is found in Fig. 3.

Table 4 summarizes the agreement of P(SIS) and P(visual) in the subgroup with CAD-RADS 1 or 2, which is of special interest because suggested recommendations of CAD-RADS 2.0 are influenced by the P score in this case. When using P(visual) instead of P(SIS), there were 11/87 cases of change of suggested recommendations (12.6%).

**Table 4:** Reclassification table for patients with CAD-RADS 1 and 2 (n = 87), comparing P scores based on segment involvement score (SIS) and on visual estimate. Boxes signifying the number of patients with change of suggested recommendation according to CAD-RADS 2.0 (P1 and 2 vs P3 and 4) are marked in red. In this case there were 11/87 cases of change of suggested recommendations (12.6%).

## 4 Discussion

Above all the current study shows that plaque burden estimates based on different methods should not be used interchangeably. The level of agreement between the three evaluated methods is moderate to substantial, pointing to the same general direction of findings, but also to differences in detail. When reporting plaque burden, the method used to estimate it should be clearly stated to avoid misinterpretation.

While the consequences of suggested clinical recommendations of CAD-RADS 2.0 are difficult to assess, the current study also shows that the plaque burden estimate method can affect these recommendations in a subgroup of patients with CAD-RADS 1 or 2

(20.6% of reclassification when using SIS instead of CAC). Further evaluation of the prognostic value of the P score is necessary, as the current study focuses on method agreement and does nor provide prognostic validation.

The results on method agreement are not particularly surprising because in essence the three methods measure different things. While SIS and visual estimate aim to be more comprehensive, calcium scoring only focuses on calcified plaques. SIS describes plaque involvement in more detail than visual estimate. To our knowledge, this is the first study to compare these three methods. In particular, the visual plaque estimate method as defined by CAD-RADS 2.0 was first evaluated in this study.

In contrast to inter-method agreement, the interrater agreement of methods evaluated in the current study has been previously studied by several authors. In this regard our results are similar to earlier work. One such example is the interrater reliability of SIS, which was shown to be very good in our study (κ = 0.82). The interrater reliability of SIS was previously evaluated by Hoffmann et al. [18] who also found it to be near perfect (κ = 0.88). Similarly, Ferencik et al. [19] found near-perfect levels of interrater agreement regarding SIS (κ = 0.85).

As CAC scoring is completely automated and very well validated, we did not perform an additional evaluation of this method's repeatability. It has already been studied by several authors before, with very good results. For example, in regard to Agatston scores, Ohnseorge et al. [20] found a minimum median variability of 9%. Sabour et al. [21] were also able to demonstrate very good repeatability of Agatston scores (ICC = 0.98).

The current study also confirms the results of Maroules et al., who were also able to show a good interrater agreement for CAD-RADS categories [22]. In contrast to our study (two readers), Maroules et al., whose study focused on interrater agreement, compared the readings of eight radiologists. Similarly, Basha et al. [23], in a study of 287 patients who underwent CCTA, also found an excellent interobserver agreement for CAD-RADS categories (four reviewers, intraclass correlation coefficient of 0.986). In addition, Basha et al. were also able to show an excellent agreement for CAD-RADS modifiers (ICC = 0.806).

An important argument for the use SIS is the combination of more comprehensive plaque assessment and well-established prognostic value offered by this method. In a meta-analysis of 11 studies (nearly 10 000 patients), Ayoub et al. were able to confirm SIS as a strong, independent predictor of cardiovascular events [24]. The more comprehensive nature of SIS is also expected to be helpful in younger patient populations, where clacified plaque is less common. Currently SIS is more time consuming than CAC, which is typically fully automated.

The main shortcoming of the widely used CAC is ignoring non-calcifying plaque. On the other hand, the prognostic value of this method is very well validated. In a large study of more than 23 000 patients, Mortensen et al. have shown plaque burden (measured using CAC) to be the most important prognostic factor for major cardiovascular disease events (myocardial infarction, stroke, and all-cause death) [25]. In this study, patients with comparable plaque burden were found to have comparable risk for major cardiovascular disease events, regardless of the grade of obstruction.

Due to the increasing use of CCTA in low- and intermediate risk patients, the need for standardized plaque burden assessment and the subsequent definition of preventive strategies and recommendations in this interdisciplinary field. The results of the current study contribute to a clearer understanding of the current state of plaque burden quantification in CAD-RADS 2.0.

### 4.1 Limitations

The current study has several limitations. One important limitation is the relatively small study size, partly caused by the decision to exclude patients with no signs of coronary artery disease in order to avoid overestimation of method agreement. A further important limitations is the use of only two readers. While both readers had extensive experience with CCTA, one had more experience (12 vs. 4 years), which can lower interrater agreement. The retrospective design of the study is also an important limitation.

Another limitation is the inclusion of different scan protocols in the study (step and shoot protocol, high-pitch spiral, and low-pitch spiral). The different protocols can influence image quality and thus indirectly also the detectability of plaques. In addition, the use of just one CT scanner is also a limitation. Tube current and tube voltage of CT examinations are typically not set manually but automatically selected by the scanner software. The algorithms for scan parameter selection may differ between vendors or scanner models and potentially lead to differences in image quality and thus influence plaque burden evaluation.

It can be assumed that CAC scoring is more prone to be influenced by problems of image quality than SIS because of its more quantitative nature. CAC scoring uses an attenuation threshold (≥130 HU), and the classification of voxels can change due to image noise or reconstruction artefacts. Of course smaller plaques can be overlooked on low quality CCTA scans too, thus underestimating plaque burden. But because of the categorical nature of SIS, this influence is expected to be less pronounced. A recent article by Khan et al. [26] has shown SIS to better correlate with AI‑derived plaque burden than CAC does when image quality varies.

### 4.2 Future directions

While the results of the current study provide an argument for the methods of plaque burden assessment provided by CAD-RADS 2.0,

we should be mindful of the fact that method agreement is just one relevant characteristic of these methods.

A major flaw of all three methods evaluated in this study is the lack of information on plaque composition, which is an important factor for major cardiac events. While CAD-RADS 2.0 provides for the modifier HRP to denote high-risk plaque, it denotes only its presence but not its extent. Features of HRP include low-attenuation plaque, positive remodeling, spotty calcifications, and the napkin-ring sign.

The extent of low attenuation plaques (non-calcified plaque with an internal Hounsfield Unit value of ≤ 30, indicating a lipid-rich core) is a very important independent prognostic factor. Williams et al. have shown low-attenuation plaque (measured using standardized semiautomatic software and given as a percentage of the plaque volume) to be the most important risk factor for fatal or nonfatal myocardial infarction [27].

Similarly, a multicentric study by Lin et al. (median follow-up of 4.7 years) was able to demonstrate that patients with a low-attenuation plaque burden of 4% or higher had a 2.5-times increased risk of myocardial infarction compared with patients with a burden below 4% [28].

The studies mentioned above underline the need for improved methods of plaque burden estimate. This is also already implied in the original CAD-RADS 2.0 paper, which states that plaque quantification is preferable but currently not standardized and not available in many institutions [9].

Chances are good for these problems to be overcome thanks to AI-enabled quantitative coronary plaque analysis (AI-QCPA), which is rapidly developing in recent years.

AI-based plaque quantification methods have demonstrated strong agreement with intravascular ultrasound (IVUS) [29] and with human readers [30], and some have even already been approved by the FDA.

### 4.3 Conclusions

Results for plaque burden in CAD-RADS 2.0 are clearly influenced by the choice of assessment method. When reporting plaque burden in CAD-RADS 2.0, the method of assessment should be unequivocally named, as the three proposed methods are not interchangeable and describe different aspects of plaque burden.

## CRediT authorship contribution statement

**Felix Matthias Heidrich:** Writing – original draft, Methodology, Investigation. **Stefanie Jellinghaus:** Writing – original draft, Methodology, Investigation. **Ralf-Thorsten Hoffmann:** Writing – review & editing, Supervision. **Axel Linke:** Writing – review & editing, Supervision. **Krzysztof Nocon:** Writing – review & editing, Formal analysis, Conceptualization. **Suela Mema:** Writing – review & editing, Visualization, Investigation. **Ivan Platzek:** Writing – original draft, Investigation, Formal analysis, Data curation, Conceptualization.

## Ethical statement

This research presents an accurate account of the work performed, all data presented are accurate and methodologies detailed enough to permit others to replicate the work.

This manuscript represents entirely original works and or if work and/or words of others have been used, that this has been appropriately cited or quoted and permission has been obtained where necessary.

This material has not been published in whole or in part elsewhere.

The manuscript is not currently being considered for publication in another journal.

That generative AI and AI-assisted technologies have not been utilized in the writing process or if used, disclosed in the manuscript the use of AI and AI-assisted technologies and a statement will appear in the published work.

That generative AI and AI-assisted technologies have not been used to create or alter images unless specifically used as part of the research design where such use must be described in a reproducible manner in the methods section.

All authors have been personally and actively involved in substantive work leading to the manuscript and will hold themselves jointly and individually responsible for its content.

## Funding

This research did not receive any specific grant from funding agencies in the public, commercial, or non-for-profit sectors.

## Declaration of Competing Interest

All authors declare that there are no conflicts of interest.
